# Der Speichel in physiologischer, diagnostischer und therapeutischer Beziehung

**Published:** 1847-01

**Authors:** 


### Art. VII.

1. *Der Speichel in physiologischer, diagnostischer und therapeutischer Beziehung.* Eine mit Anmerkungen vermehrte Bearbeitung, nach S. Wright, m.d., &c.—*Wien,* 1844. 8vo, pp. 213.

2. *Het Speeksel uit een, physiologisch, diagnostisch, en therapeutisch oogpunt beschouwd,* door S. Wright, m.d. Naar de Hoogduitsche bearbeiding van Dr. S. Eckstein, vertaald en met aanteekeningen voorzien door F. Rienderhoff.—*Amersfoort,* 1846. 8vo, pp. 220.

The fact of a work being translated into two different languages, shortly after the publication of the original, suffices to show, at least, the interest attaching to the subject, and may be regarded as a fair indication of its importance, as well as of the satisfactory way in which the subject has been handled by the author. Admitting this, it may seem strange that such a work, coming out in our own country and in our own tongue, should have remained unnoticed by us until we received it in the shape into which it has been put by our German and Dutch brethren. The truth is, that Dr. Wright's treatise has never been published in this country in the form of a substantive book at all. It appeared originally in the 'Lancet' journal, as a series of papers; and it is from these that the translations now before us have been made.

As we doubt not that we shall have another opportunity of considering Dr. Wright's labours in a yet more perfect form, we shall, on the present occasion, confine ourselves to giving a mere analytical outline of the work, without any comments, critical or otherwise. And, in doing so, we shall—as well for our own case as for the sake of perfect accuracy—make use of the author's original diction, as given in the 'Lancet,' instead of retranslating the passages from the volumes on our table.

Dr. Wright commences by tracing the derivation of the term saliva. After quoting a variety of opinions upon it he concludes that, "the word is probably derived both from the Greek and Latin languages—the one being expressive of the manner in which the fluid is secreted and discharged; the other bearing an allusion to its saline constituents. Hence the Irish term *silim,* which signifies to drop or to distil; and the Welsh *haliw,* from *hâl,* salt." The author then passes on to consider the chemical history, properties, and composition of the saliva in its healthy state. Having quoted the authorities preceding him, he details his own process for the analysis of saliva. To obtain this fluid unmixed with buccal mucus is a matter of the greatest consequence, and not always easy of accomplishment.

"By moving the lower jaw and the tongue, as in the ordinary way of procuring saliva, this fluid necessarily becomes united with some mucus from the lining membrane of the mouth. For accurate investigation, it is necessary to obviate this intermixture, and it is best effected, according to my own observation, in the following way. The mouth should first be washed with cold water, and then, having depressed and fixed the lower jaw, the fauces are to be tickled with a feather, sufficiently to excite nausea, without the risk of vomiting. The saliva will sometimes run from the mouth in a stream, and generally it may be obtained in sufficient quantity for examination, and free from extraneous matter. A little cayenne pepper, or any similar irritant, applied to the sublingual gland, will excite an abundant secretion; but, as I shall hereafter show, the saliva obtained by these means contains more than its natural proportion of free alkali."

Dr. Wright's analytical process has been repeated by Budge and Lehmann, and for the most part confirmed by them. (See Mr. Paget's Report on the Progress of Anatomy and Physiology for 1842-3. Brit. and For. Med. Rev. January 1844. p. 258, note.) He states the composition of healthy saliva to be, as follows—

| | |
|---|---|
| Water | 988·1 |
| Ptyalin | 1·8 |
| Fatty acid | ·5 |
| Chlorides of sodium and potassium | 1·4 |
| Albumen, with soda | ·9 |
| Albuminate of soda | ·8 |
| Phosphate of lime | ·6 |
| Lactates of potass and soda | ·7 |
| Sulphocyanide of potassium | ·9 |
| Soda | ·5 |
| Mucus, with ptyalin | 2·6 |

Whether there be such a principle as ptyalin, and what it is, are questions about which chemists have long been puzzling themselves. There can be no doubt that what Berzelius called ptyalin, was neither more nor less than albumen. (Dr. Golding Bird, Lond. Med. Gazette 1840-41. No. 43, pp. 643-4.) It is more than probable, also, from the manner in which Simon, Mialhe, and others separate their ptyaline, that it is either albumen or some other proteine-compound. Dr. Wright's ptyalin would appear to be different from that of other chemists, and is obtained in a totally different way, as we learn from the following detail.

"First, pass saliva through ordinary filtering paper, and, after filtration shall have been completed, secondly, exhaust the residue with sulphuric ether; the ethereal solution contains a fatty acid and ptyalin. It is to be allowed to evaporate spontaneously; and thirdly, the residue left by evaporation is to be placed upon a filter and acted upon by distilled water, which dissolves the ptyalin and leaves the fatty acid. If the aqueous solution be evaporated to near dryness, the "salivary matter" will be obtained in a pure state. Ptyalin, as thus prepared, is a yellowish-white, adhesive, and nearly solid matter; neither acid nor alkaline, readily soluble in ether, alcohol, and essential oils, and more sparingly soluble in water. It alone possesses the characteristic odour of saliva; it is unaffected by galvanism, and by most of the reagents which coagulate albumen; it is abundantly precipitated by sub-acetate of lead and by nitrate of silver; feebly so by acetate and nitrate of lead and tincture of galls; uninfluenced by bichloride of mercury and strong acids: the latter considerably heighten its proper odour and impair its solubility, whilst alkalies render it more soluble and give it the smell of mucus. Moderate heat, and oxygen gas also increase its odour; but intense heat or cold diminishes or entirely destroys it. At a suitable temperature, ptyalin may be preserved for any length of time without risk of decomposition. The salivary fluid from which ptyalin has been removed by filtration possesses a sickly mucous smell, decomposes much sooner than ordinary saliva, and in the process of decay invariably evolves ammonia. If this fluid be heated, the mucous smell will be increased until the evaporation shall have been continued nearly to dryness; when a slight salivary odour may be recognized, due to a portion of ptyalin being liberated from the mucus with which it was previously in combination."

The ptyalin thus obtained, and thus described, by Dr. Wright, bears no resemblance to the proteine-compounds of other chemists: its possessing the characteristic odour of saliva, an odour which no other solid or fluid has in common with this secretion, strengthens greatly its claim to be considered as valid: still the question is yet *sub judice,* and we hope that the investigations to decide it are not likely to be much longer delayed.

The physiological and medical history of saliva are next treated of. After quoting divers authorities from Theophrastus downwards, in illustration of the superstitious and other applications of saliva, Dr. Wright enumerates a variety of experiments of his own, in proof of the physiological activity of this secretion. Of fourteen experiments, we select the two following as types of the whole. The quotation is long; but the great importance of the subject justifies it; and we are sure our readers will not think it tedious.

"Four drachms of slightly alkaline saliva, sp. gr. 1·010, were injected into the right external jugular of an old mongrel dog. Immediately after the fluid had passed, the animal uttered a loud yell and struggled violently; the heart palpitated with vehemence, and respiration became very hurried and irregular. When six minutes had elapsed, and the severe effects had subsided, other four drachms of saliva were injected. The heart's action again became so quickened that I was unable to number its beats; the pupil was contracted; the abdominal muscles underwent a strong spasm; and there was slight convulsion of the whole frame. At the expiration of ten minutes, the injection was repeated; it had the effect of increasing, but not remarkably, the action of the heart and lungs; the spasm of the abdominal muscles returned, and a quantity of bile and frothy mucus was ejected by vomiting. When thirteen minutes and a half had elapsed, an abundance of turbid urine, and fæces mixed with blood, were passed: severe tenesmus succeeded, accompanied also by slight priapism. At the expiration of twenty-five minutes, when the system was comparatively calm, the pupil a little dilated, but sensible to light, and the heart beating seventy-two strokes per minute, I injected the remaining four drachms of saliva into the vein. The symptoms which attended the first injection instantly recurred, but with increased violence, and continued, with trifling remissions, for nearly four minutes, after which time their severity subsided. At the end of forty minutes, there was slight convulsion of the whole frame; an offensive slimy dejection was passed, to all appearance involuntarily; and shortly afterwards, about half a pint of bloody urine escaped in a similar manner. When three hours had elapsed, the animal seemed to be tolerably calm and comfortable; he ate a little meat, and lapped milk and water: he was then left for the night.

It was observed on the following morning that he had made a great quantity of water, and that he had been purged and vomited several times. He now looked drowsy and stupid; his eye was dull, watery, and injected; he was disinclined for sport and exercise; he ate little, but drank abundantly; respiration natural; pulse 86.

In three or four days the animal recovered his usual hearty and lively habits, and little notice was taken of him until the morning of the fifteenth day succeeding the experiment, when he was observed to look drowsy and dejected; his eyes were peculiarly downcast and inflamed; he refused to stir when called, and when approached, he uttered a growl expressive of anxiety and anger; his nose was dry and warm; paws cold; respiration irregular and quick; pulse 94. He lapped water or milk, but refused solid food. He continued in this state through out the day, passing one very offensive, dark, slimy, stool, and voiding at several efforts a great quantity of turbid bilious urine.

On the following morning, the symptoms of the previous day were much aggravated; the dog growled and snapped at everything, living and lifeless, that approached him. My assistant in alarm ran away, and the other attendants being terrified at the dog's madness, deserted me also, and I was consequently left to manage him alone. By unobservedly seizing him at the back of the neck with one hand, and grasping him tightly, I was enabled to raise his lips with the other, when I observed that his mouth was filled with foam, and that his gums and cheeks were much swollen and inflamed; his nose was very dry and hot; paws cold and tender; respiration interrupted with frequent sighing; pulse 106. So long as I held him in my grasp, he seemed to be quite docile and contented, but the moment I loosed my hold, he ran furiously at me, and but for a strong chain which secured him to the wall, I have no doubt I should have suffered from his bites. In a few hours, the froth began to distil from his mouth; he was tortured with thirst, and lapped water eagerly, and without dread; indeed he plunged his mouth and face into the cold liquid, as if to relieve the heat and inflammation which troubled him; with the same intention he licked the cold wall and floor, but he would not touch any warm body, nor would he lap tepid water. He was remarkably irritable and restless, and when not snapping at objects that approached him, was constantly turning about, or chewing the sand and straw that were near. He was seen in the afternoon of this day by Mr. Bowker, surgeon, and by several other scientific friends, all of whom were decidedly of opinion that he was the subject of madness. In the evening, his restlessness somewhat abated, and he lay moaning in a husky voice, occasionally altering the position of his head, as if anxious to sleep. Thirst and salivation were diminished; eye dull and glassy, pulse 64, nose dry, extremities cold. He continued in this state, with increasing weakness and somnolency, until about five o'clock on the following morning, when, after a few struggles and signs of suffocation, he died.

*Sectio cadaveris, six hours after death.* The limbs were remarkably rigid, but the blood was everywhere uncoagulated, and presented scarcely any distinction between arterial and venous. No ptyalin could be found in it. The right cavities of the heart were gorged with blood; the left auricle was also full, and the left ventricle empty. The lungs were moderately crepitous, and unusually vascular; the air-cells and bronchi contained an abundance of mucus. The lining membrane of the trachea was vascular, and the redness extended to the membrane of the mouth. The gullet was also unnaturally florid, but the stomach and intestines presented nothing unusual. The stomach contained a quantity of straw, sand, and coal. The other viscera were natural. The brain exhibited venous congestion upon its surface, and a little bloody serum at its base; but in other respects it was healthy."

The second case is of the same interesting character.

"Six drachms of nentral saliva, sp. gr. 1·008, were introduced at three separate injections into the right common carotid of a mongrel dog. Each injection was followed by an extraordinary increase in the heart's action; hurried, irregular respiration, and general convulsion. The symptoms closely resembled those detailed in a similar experiment, especially in the inability of the animal to walk in a straight direction, and his consequent movement in a circle: the inclination was always towards the vessel which had received the injection. The stage of excitement lasted for five hours, during which time the animal passed an abundance of urine, vomited, and was purged several times. At the end of six hours, slight coma supervened; respiration was deep and stertorous; heart's action slow and laboured; sensibility very diminished.

On the following day there was considerable reaction, and the animal manifested strong signs of irritability and excitement. On the evening of this day, I was called from home, and was unavoidably absent for a week. On my return, I learnt that, on the second day succeeding the experiment, the dog became calm and docile, ate and drank very well, and appeared not to suffer either pain or inconvenience of any kind. In this state he continued until the morning of the eighth day, when, on being visited with his breakfast, he flew at the servant, who narrowly escaped him. The door of the place in which he was kept was divided horizontally in two, so that by shutting the bottom half, he could be conveniently watched over it. He was described to me as frothing considerably at the mouth, and appearing very fierce in the eyes, which were deeply reddened. He wandered about incessantly, chewing straw or sand, or lapping a little water; but he refused all kinds of solid food. He was shortly left to himself, when he began to gnaw the bottom of the door, which he finally demolished to an extent sufficient for his escape. On my return home in the evening of this day, I discovered him in the middle of a field contiguous to the house, surrounded by half a dozen men, who, with sticks, forks, and spades, were variously endeavouring to get him back into the stable. It was sufficiently ludicrous to see such an amount of human srength and ingenuity successfully combated by a single brute; but the men were in thorough trepidation from the manifest signs of madness which the dog exhibited. He snapped furiously at everything that approached him, and would occasionally pursue one of his opponents, until, tired by the effort, he was compelled to stop for breath. When I saw him he was staring wildly, and a quantity of frothy saliva was distilling from his mouth: the anterior part of his body was covered with this foam. I put a strong glove upon my right hand, and whilst the dog was engaged in snapping at a stick held before him, I caught him by the back of the neck. He struggled violently at first, and seemed to be choking, but finding resistance useless, he became perfectly quiet and composed. From an experience of the treachery of the animal in the eighth experiment, I did not venture upon loosing my grasp until having examined the state of the eyes and mouth; both which I found to be unusually vascular, and the pulsations of the heart were 140 per minute, when I had a collar with a strong chain fastened round the dog's neck, after which he was reconducted to his stable.

"He was visited again in an hour, but there was no observable decrease of irritability or restlessness; he snapped at anything that came in his way, and was incessantly changing his position. He lapped a little water, but the only solid matter he would chew were fragments of sand and coal. On the following morning he was much in the same state, but less inclined to bite: his mouth was still very frothy, and his eye deeply reddened; respiration rather stertorous; pulse 98. The irritability and restlessness increased towards evening; he would allow nothing to approach him without snapping at it; he was constantly engaged in licking the cold wall, or chewing straw, sand, or coal; or dragging himself upon his belly over the rough ground. On the morning of the tenth day he was somewhat improved; he ate a little meat, and did not snap unless suddenly roused; the salivation was less, and the eye appeared to be brighter; there was no stertor, and the pulse was 84.

From this time the signs of madness diminished, and the dog seemed to be improving in health and strength until a fortnight had elapsed, when there occurred a most offensive discharge from his nose and ears; it was greenish-yellow in colour, excessively fetid, acrid and corrosive, for it excoriated the parts over which it trickled; and finally caused the entire of the nose and one ear to slough away. In a few days the dog became quite blind and deaf, though he did not diminish in strength, and he ate very heartily of meat which was plentifully supplied him. He did not appear to suffer any pain, and was seemingly very quiet and contented. He continued in this state for more than three weeks longer, at the end of which time I was compelled to leave home: I learnt, however, that ulcers subsequently appeared in different parts of his body, and were succeeded by gangrene of the extremities, of which he died. The body was in a state of putrefaction before death. This animal was several times seen by my friends Drs. Hutchinson and Taylor, and Mr. Massey and Mr. Thompson, surgeons of Nottingham."

To determine whether the mere animal matter of the saliva had any share in producing the above phenomena Dr. Wright performed the following experiments.

"I injected a drachm of isinglass dissolved in two ounces of water, into the carotid artery; a little temporary excitement was the consequence, but the animal suffered no further inconvenience.

"I injected a drachm of pure mucus, diffused through an ounce and a half of water, into the jugular vein; the heart was a little quickened, and respiration was correspondently hurried, but the effects completely subsided in twenty minutes.

"I injected the entire white of an egg, diffused through two ounces of water, into the left common carotid of a dog. It produced considerable cerebral excitement, which was succeeded by drowsiness and feebleness of the limbs that continued for several hours; but the symptoms were very mild, and their duration inconsiderable."

The bearing of these experiments on the momentous question of the etiology of hydrophobia will occur to every reader; and we think the author uses a due discretion in speaking doubtfully on the subject.

"Concerning the production of canine madness by the injection of saliva into the blood, it is not now my intention to speak. It will, however, be sufficiently evident from the experiments already cited, that saliva is capable of exerting a very marked influence upon the brain and nervous system. The spasm, convulsion, and coma which were consequent upon the introduction of saliva into the arteries and veins, are conclusive proofs of its activity; whilst the absence of all such symptoms on the injection of the other animal fluids into the circulatory system demonstrates that, not to any physical or mechanical influence, but to a peculiar property inherent in itself, is saliva indebted for the manifestation of its physiological action."

Dr. Wright next proceeds to consider the digestive properties of saliva. He finds that its alkalinity varies, in the healthy subject, according as the stomach may be excited by stimulants, or by the presence of acidity—proportionately to these things is the alkalinity of the saliva increased.

"I tested the saliva of a dog, and found it moderately alkaline. I then injected into his stomach, through an elastic catheter, an ounce of French brandy diluted with an equal quantity of water. In three minutes, the alkalinity of the saliva was doubled, and at the end of six minutes its strength was four or five times greater than at first. At the expiration of half an hour it had returned to its original state of alkalinity. I repeated this experiment several times upon other animals, and always with the same results.

"I was led to make an extensive series of observations upon this subject, from noticing in my own case the effect of spitting after a full meal. In such instances I never failed to have an abundance of acidity, with much pain in my stomach, *and a correspondent alkalinity in my saliva.* By taking a dose of carbonate of soda, which was immediately succeeded by the rapid disengagement of carbonic acid gas, *my saliva would return to feeble alkalinity in a few minutes, and often in a few seconds.* These observations, at a sad cost of health and strength, I made upon myself nearly one hundred times. Frequent attacks of dyspepsia, to which I was once happily a stranger, painfully remind me of the injury I sustained in the course of my investigations. I once spat two hundred and fifty drachms of saliva in one week, and from the nature of my experiments I was often compelled to spit directly after dinner: in that seven days I lost eleven pounds in weight, and was much weakened and emaciated."

After a variety of experiments, tending to show the digestive action of saliva upon starch and other substances, Dr. Wright enumerates the two following, amongst several others, as more directly proving the important part saliva serves in the common function of digestion.

"Eight ounces of lean, raw beef, and eight ounces of bread, were beaten to a pulp with ten ounces of water, and the whole was then injected through the tube of a stomach-pump into the stomach of a healthy terrier dog weighing 14lbs. The animal had fasted for twenty hours previously. Immediately after the injection the gullet was tied. The saliva was tested at this time, and was observed to be *feebly alkaline. In half an hour its strength of alkalinity was at least doubled;* the *quantity* of secretion was also much augmented. *The alkaline reaction of the saliva increased, till, at the end of three hours, it contained* 3·14 *per cent of alkali.* At this time the animal was destroyed by the introduction of air into his jugular vein. The stomach smelt excessively sour, and its mucous membrane was deeply reddened; the shade was at least three or four times deeper than that which is observable in ordinary digestion. The food, especially the fibres of the meat, seemed to be little altered, and except being incorporated with an abundance of mucus, presented almost the identical appearance of the original mixture.

"Eight ounces of lean, raw beef, and eight ounces of bread, were beaten to a pulp with ten ounces of alkaline saliva, and the mass was subsequently injected into the stomach of a healthy bull-terrier dog weighing 13lbs. He had taken no food for twenty hours previously. The gullet was tied as before. On testing the animal's saliva, it was found to be moderately alkaline. *At the end of half an hour, the secretion was scarcely altered either in character or quantity.* When two hours had elapsed, the mouth was very frothy, but the saliva was little changed. At the end of three hours the mouth was still frothy, and *the saliva contained only* ·89 *per cent. of alkali.* The animal was now killed by the injection of air into his jugular vein. The contents of the stomach were not particularly acid, nor was the mucous membrane more vascular than in ordinary digestion. The food was reduced to a perfectly homogeneous pulp, and not a trace of fibre could be found in it."

The following are the "Inferences" deduced by Dr. Wright, from his experiments concerning the digestive properties of saliva.

"1. Saliva has the power of modifying, and to a certain extent, of digesting vegetable and animal substances.

2. It has a more powerful action upon vegetable than upon animal matters.

3. The healthy digestive action of saliva is always attended with the evolution of lactic acid.

4. Filtration, or boiling, diminishes the digestive powers of saliva, but does not destroy them.

5. Exposure of the saliva to atmospheric air, for a moderate length of time, does not materially weaken its digestive powers; but they are enfeebled in the ratio of the putrescency of the secretion.

6. Oxygen gas assists the digestive action of saliva, but is not essential to it. Carbonic acid gas impairs this action in a mild degree, and hydrogen and nitrogen gases weaken it very considerably.

7. Acids or alkalies added to saliva, diminish or destroy its digestive properties.

8. The presence of saliva in the stomach is essential to healthy digestion.

9. The digestive action of saliva is not possessed in any efficient degree by animal mucus, acids, alkalies, or alkaline salts."

The services performed by saliva in the animal economy he divides into active and passive. The former are—1st, to stimulate the stomach and excite it to activity by contact; 2d, to aid the digestion of food by a specific action upon the food itself; 3d, to neutralize any undue acidity in the stomach, by supplying a proportionate alkali. The latter, 1st, to assist the sense of taste; 2d, to favour the expression of the voice; 3d, to clear the mucous membrane of the mouth, and moderate thirst.

The pathology of saliva concludes the work, and constitutes, indeed, the greater part of it. Dr. Wright divides the pathological varieties of saliva into the following: "Deficient saliva; redundant saliva (*α* spontaneous,

β excited); fatty saliva; sweet saliva; albuminous saliva (α transparent, β white); bilious saliva; bloody saliva; acid saliva; alkaline saliva (α fixed alkali, β ammoniacal); calcareous saliva; saline saliva; puriform saliva; fetid saliva; acrid saliva (α per se, β from foreign matters); coloured saliva; frothy saliva; urinary saliva; gelatinous saliva." Each of these is treated at length, as regards its physical and chemical characters, its pathognomonic relatives, and its bearings upon diagnosis. Depraved states of the saliva, Dr. Wright thinks are often dependent upon idiopathic disordered function of the salivary glands, and that functional error of the gastro-intestinal apparatus occurs from the ingestion of this depraved fluid. In these cases, and in others in which the saliva is deficient, Dr. Wright lays great stress on the use of stimulant gargarisms, and epispastic or irritant applications in the neighbourhood of the salivary glands. In excessive secretion of the salivary fluid, he advises sedative and astringent gargarisms.

Without departing from our avowed intention of refraining at present from any formal criticism of Dr. Wright's production, we cannot be so ungracious as to dismiss it from our notice without one word expressive of our opinion of its value as a contribution to physiology. Even in the meagre outline of the treatise given above, the reader will discover its great interest and importance, and will receive it as conclusive evidence of the ingenuity, zeal, industry, and philosophical spirit of the author.

---

